# Identification of Antibiotics in Surface-Groundwater. A Tool towards the Ecopharmacovigilance Approach: A Portuguese Case-Study

**DOI:** 10.3390/antibiotics10080888

**Published:** 2021-07-21

**Authors:** Paula Viana, Leonor Meisel, Ana Lopes, Rosário de Jesus, Georgina Sarmento, Sofia Duarte, Bruno Sepodes, Ana Fernandes, Margarida M. Correia dos Santos, Anabela Almeida, M. Conceição Oliveira

**Affiliations:** 1 Agência Portuguesa do Ambiente (APA), Rua da Murgueira, 9, Zambujal, Alfragide, 2610-124 Amadora, Portugal; paula.viana@apambiente.pt (P.V.); ana.rita@apambiente.pt (A.L.); rosarioj@apambiente.pt (R.d.J.); 2 Autoridade Nacional do Medicamento e Produtos de Saúde, I.P. (INFARMED), Avenida do Brasil, 53, 1749-004 Lisboa, Portugal; 3 Faculdade de Farmácia, Universidade de Lisboa, Avenida Prof. Gama Pinto, 1649-004 Lisboa, Portugal; bsepodes@ff.ulisboa.pt; 4 Laboratory of Systems Integration Pharmacology, Clinical & Regulatory Science, Research Institute for Medicines (iMED.ULisboa), 1600-277 Lisboa, Portugal; 5 Laboratório de Análises, Instituto Superior Técnico, Universidade de Lisboa, Av. Rovisco Pais, 1049-001 Lisboa, Portugal; gsarmento@tecnico.ulisboa.pt (G.S.); anafernandes@tecnico.ulisboa.pt (A.F.); mcsantos@tecnico.ulisboa.pt (M.M.C.d.S.); 6 Centro de Investigação Vasco da Gama (CIVG), Departamento de Ciências Veterinárias, Escola Universitária Vasco da Gama (EUVG), Campus Universitário de Lordemão, Av. José R. Sousa Fernandes 197, 3020-210 Coimbra, Portugal; s.cancela.duarte@gmail.com; 7 REQUIMTE-LAQV, Laboratório de Bromatologia e Farmacognosia, Faculdade de Farmácia da Universidade de Coimbra, Polo III, Azinhaga de Santa Comba, 3000-548 Coimbra, Portugal; 8 Centro de Química Estrutural, Instituto Superior Técnico, Universidade de Lisboa, Av. Rovisco Pais, 1049-001 Lisboa, Portugal; conceicao.oliveira@tecnico.ulisboa.pt; 9 Coimbra Institute for Biomedical Imaging and Translational Research (CIBIT), University of Coimbra, 3000-548 Coimbra, Portugal

**Keywords:** antibiotics, surface- groundwater, POCIS, ecopharmacovigilance, cilastatin, tazobactam, abacavir

## Abstract

Environmental monitoring, particularly of water, is crucial to screen and preselect potential hazardous substances for policy guidance and risk minimisation strategies. In Portugal, extensive data are missing. This work aimed to perform a qualitative survey of antibiotics in surface- groundwater, reflecting demographic, spatial, consumption and drug profiles during an observational period of three years. A passive sampling technique (POCIS) and high-resolution chromatographic system were used to monitor and analyse the antibiotics. The most frequently detected antibiotics were enrofloxacin/ciprofloxacin and tetracycline in surface-groundwater, while clarithromycin/erythromycin and sulfamethoxazole were identified only in surface water. The detection of enzyme inhibitors (e.g., tazobactam/cilastatin) used exclusively in hospitals and abacavir, a specific human medicine was also noteworthy. North (Guimarães, Santo Tirso and Porto) and South (Faro, Olhão and Portimão) Portugal were the regions with the most significant frequency of substances in surface water. The relatively higher detection downstream of the effluent discharge points compared with a low detection upstream could be attributed to a low efficiency in urban wastewater treatment plants and an increased agricultural pressure. This screening approach is essential to identify substances in order to perform future quantitative risk assessment and establishing water quality standards. The greatest challenge of this survey data is to promote an ecopharmacovigilance framework, implement measures to avoid misuse/overuse of antibiotics and slow down emission and antibiotic resistance.

## 1. Introduction

Antibiotics are a critical component of human and veterinary modern medicine, developed to produce desirable or beneficial effects on infections induced by pathogens. Like most pharmaceuticals, antibiotics tend to be small organic polar compounds, generally ionisable, ordinarily subject to a metabolism or biotransformation process by the organism to be eliminated more efficiently [1,2]. The excretion of these compounds and their metabolites occurs mainly through urine, faeces, or a combination of both. The primary sources for antibiotics contamination are domestic, urban, hospital, industrial wastewater, aquaculture, and intensive livestock farming [3]. As the existing sewage treatment plants cannot efficiently remove them, they may enter the natural surface-ground water in detectable amounts. Antibiotics, apart from other contaminants, can disturb the micro-ecosystem by expansion of resistance to them [4]. The study of environmental antibiotic resistance dissemination is currently relevant to the scientific community [5,6]. The assessment of antibiotics in water has been possible due to the increasing sensitivity/resolution of analytical methods, such as ultra-performance liquid chromatography/quadrupole time-of-flight mass spectrometry (UHPLC-QqTOF-MS) and the long adsorption time of substances in passive samplers [7]. Since 2006, marketing authorisation procedures for both human and veterinary medicines must include an environmental risk assessment that comprises a prospective exposure assessment, underestimating the possible impact and the occurrence of antibiotics after years of consumption. Ultimately, the potential risk may not be correctly anticipated. It becomes urgent to generate new data, mainly to refine exposure assessments. As much as the specificities of each member state should be considered this issue has become one of the European community’s main concerns [8]. 

The strategies against water pollution are provided in the Water Framework Directive [9] and the Directive on Environmental Quality Standards that set environmental quality standards (EQS) for the substances in surface waters and confirm their designation as priority or priority hazardous substances [10]. Evidence of potential impacts and persistence of pharmaceuticals on aquatic organisms have been documented, enforcing the inclusion of antibiotics as a candidate in the EU List of Priority Substances [11].

To identify and monitor sources of emerging pharmaceuticals in surface water, the guidance document “*On Surface-water Chemical Monitoring*” encourages the use of passive sampling methodology as a risk-based screening tool to evaluate the presence of contaminants [12]. Passive sampling is a monitoring technique that can provide a representative picture of levels of pharmaceuticals and their metabolites [13]. Polar organic chemical integrative sampler (POCIS) is a methodology based on the passive diffusion of pharmaceuticals and their metabolites from the aquatic environment. In contrast, this integrative passive sampler enables the estimation of pharmaceuticals/metabolites contaminant concentrations over extended sampling periods to grab or composite sampling strategies. Thus, the preconcentration of contaminants leads to an increase in the ability to detect trace concentrations [7].

The detection of antibiotics and their metabolites in water is crucial to screen and preselect potential hazardous compounds for policy guidance and the implementation of risk minimisation strategies. In Portugal, data are missing, and the comparison between detection and consumption remains unexplored. This work aims to perform a qualitative survey of antibiotics and their metabolites in surface- groundwater. It seeks to reflect the current demographic, spatial, drug consumption, and drug profile on an observational period of 3 years in Portugal. The greatest challenge of this survey data will be to promote the ecopharmacovigilance framework development shortly to implement measures for avoiding misuse/overuse of antibiotics and slow down emission and antibiotic resistance.

## 2. Results

### 2.1. Frequency of Detections: Antibiotics/Enzyme-Inhibitors and Abacavir in Surface-Groundwater

During the screening framework beyond the antibiotics/enzyme-inhibitors, the antiviral abacavir was detected. Therefore, given the relevance of this compound, it was included in the present study. Although enzyme inhibitors belong to the antibiotic group, their specific pharmacological properties and detection were sorted apart. In the present study, antibiotic metabolites were not detected in the water bodies.

#### 2.1.1. Antibiotics/Enzyme-Inhibitors and Abacavir in Surface-Water

Fifty detections were found in all catchment areas in surface water, which corresponds to 15 different active substances: 12 antibiotics, two enzyme inhibitors, and one antiviral. The number of detections per sampling station ranged from 0 to 7 different active substances. The Ave river—Prazins (Santo Tirso) and Serzedelo I and II (Guimarães) as well as Ria Formosa—coastal water (Faro and Olhão), each one with two sampling sites, showed the most detected compounds in terms of contamination. However, spatial differences in the number of substances between the two water bodies were not evident, categorised by significant urban pressure and animal production, for instance, in the Ria Formosa aquaculture. The samples collected in the Tinto river, near Porto, the second biggest city in Portugal, showed a high frequency of detections of the aforementioned pharmaceuticals. Supplemental Tables S1 and S3 contain a detailed description of the characterisation of sampling stations and the list of all pharmaceuticals detected in surface water. The most frequently detected pharmaceutical in decreasing order were the following: abacavir 69% (9/13), ciprofloxacin, clarithromycin 46% (6/13), erythromycin 38% (5/13)) enrofloxacin, tetracycline 31% (4/13), sulfamethoxazole 23% (3/13) and amoxicillin, lincomycin, trimethoprim 15% (2/13). On the other hand, antibiotics such as azithromycin, norfloxacin, and sulfamethazine were the lowest in detection frequency 8% (1/13). Sulfamethazine was the only detected substance in the reservoir water body, which was located in rural surroundings. 

The enzyme-inhibitors demonstrated a low-frequency detection 15% (2/13). In Ave river and Ria Formosa, tazobactam has been found in a catchment area downstream of urban waste treatment plants (hospital/domestic) with equivalent numbers of inhabitants of 396,822 and 113,200, respectively.

**Figure 1:**
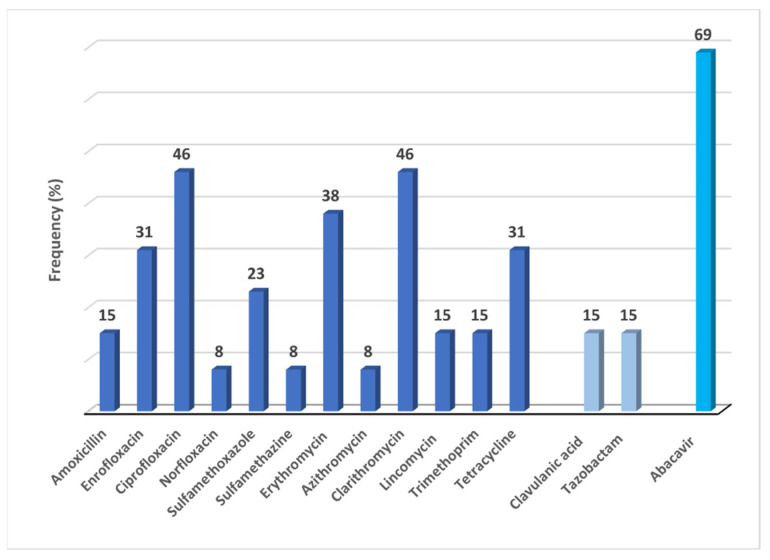
Frequency (%) of antibiotics/ enzyme inhibitors and abacavir detected in surface water.

North (Guimarães, Santo Tirso and Porto) and Algarve (Faro, Olhão and Portimão) were the regions with the most significant number of substances observed in surface water. In the North, 19 pharmaceuticals were detected (38%) against 16 in the South (32%). No pharmaceutical was detected in the two sampling stations located in the Tejo river, one near the border (Portugal/Spain) and the other in a reservoir, covering both rural areas. All surface water samples were contaminated by at least one antibiotic, while in 62% of the samples, a mixture of more than four active substances co-occurred. Figure 1 shows the frequency (%) of detected substances in surface water.

#### 2.1.2. Antibiotics/Enzyme-Inhibitors and Abacavir in Groundwater

**Figure 2:**
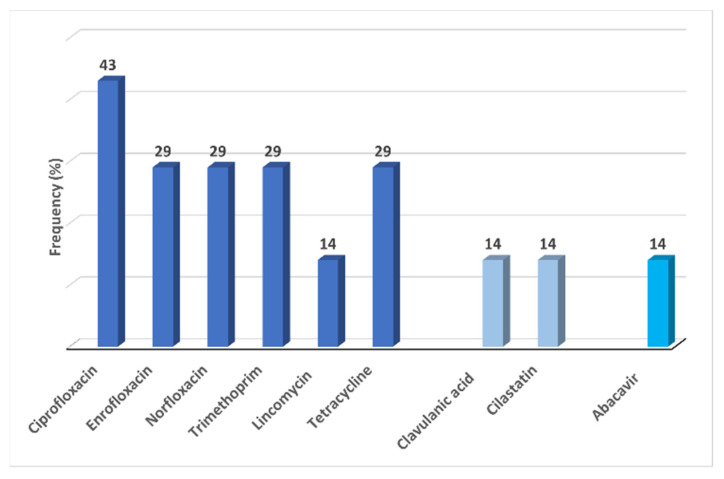
Frequency (%) of antibiotics/enzyme inhibitors and abacavir detected in groundwater.

Fifteen active substances were detected in groundwater bodies. The number of detections per sampling station ranged from one to five compounds. The most frequently identified pharmaceuticals, in decreasing order, were ciprofloxacin 43% (3/7), enrofloxacin, norfloxacin, trimethoprim, lincomycin (29% (2/7), abacavir and tetracycline 14% (1/7). The enzyme inhibitors, namely clavulanic acid and cilastatin, were detected once in an urban region located well. This catchment point showed the most significant number of pharmaceuticals. West/Tejo and Centre were the regions with the most considerable number of substances in groundwater, accounting for 43%. All groundwater samples were contaminated by at least one antibiotic. The wells contamination profile is included in Figure 2. Supplemental Tables S2 and S4 contain a detailed description of the characterisation of sampling stations and the list of all pharmaceuticals detected in groundwater.

### 2.2. Consumption of Antibiotics/Enzyme-Inhibitors and Abacavir

The annual used amount of the different therapeutic groups was markedly more significant for penicillins and tetracyclines, which alone accounted for more than 38% of each one, followed by sulfonamides (7.0%), quinolones (7.0%), macrolides and lincosamides (3.0%) as well as trimethoprim with 0.5%. 

The most representative active substances, each with more than one tonne of consumption were: tetracycline, amoxicillin, clavulanic acid, enrofloxacin, ciprofloxacin, lincomycin, sulfamethoxazole, azithromycin, and clarithromycin.

In addition to clavulanic acid (associated with amoxicillin), other detected enzyme-inhibitors, tazobactam and cilastatin, both of exclusive hospital use, showed 717 and 76 kg, respectively. They are applied in combination with piperacillin and imipenem, respectively. Regarding the different regions, it has been concluded that North and West/Tejo were the regions with the higher consuming values. Both regions presented a significant value (33%) for the abacavir. For the detected antiviral abacavir, an amount of 1458 kg has been observed.

Regarding antibiotics used in veterinary medicine, the regional amount was not available. Likewise, due to the reported missing quantity for sulfamethazine, the sulfonamides group has been matched.

**Figure 3:**
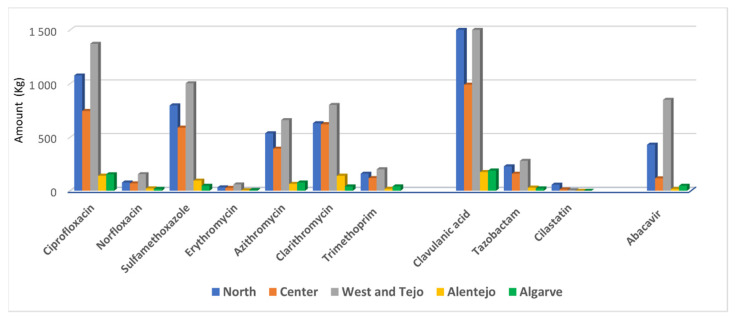
Amount of detected pharmaceuticals consumption per Portuguese region.

The estimated amounts of the consumed medicines in 2017, regarding the detected molecules used in the human primary health care sector/hospital and veterinary medicine, are displayed in Table 1. Figure 3 presents the distribution by those Portuguese regions that match the river basin districts.

### 2.3. Physicochemical Properties and Key Pharmacokinetic Features of Detected Pharmaceuticals

The detected pharmaceuticals showed high to moderate water solubility and are small ionisable molecules (MW ≤ 900 g/mol). Regarding the octanol/water partitioning coefficient (log Kow) data, macrolide antibiotics (azithromycin and clarithromycin) were the only ones that demonstrated the potential to concentrate in living organisms (log Kow ≥ 3) [14]. All the remaining antibiotics showed a relatively low log Kow and were expected to be present mainly in surface water. However, the soil mobility/adsorption detected substances exhibited different organic carbon adsorption coefficient (log Koc) values. Hence considering the soil mobility potential (0–1.7 very high; 1.7–2.2 high; 2.2–2.7 medium; 2.7–3.3 low; 3.3–3.7 slight; ≥3.7 immobile) according to McCall and adapted from Chen et al. [15], antibiotics were categorised as follows: (1) Very high mobility: tazobactam; (2) Moderate mobility: sulfamethoxazole, sulfamethazine, erythromycin, clarithromycin, trimethoprim; (3) Low mobility: amoxicillin, abacavir; (4) Slight mobility: azithromycin; (5) Immobile: enrofloxacin, ciprofloxacin, norfloxacin, tetracycline. Data for the adsorption coefficients were not available for lincomycin, clavulanic acid and cilastatin. The properties of detected antibiotics and abacavir are included in Table 2. 

Pharmacokinetic features were considered to characterise further detected molecules in the analysed water compartment—the percentage of unaltered form excreted by renal and metabolites after biotransformation are depicted in Table 3. 

The most significant number of detected substances showed a percentage of unchanged excretion higher than 40%. Renal excretion is relatively frequent; however, faecal excretion is incontestable for lincomycin (79 to 86%).

Regarding bioavailability, antibiotics show high variability among species. For instance, amoxicillin is well absorbed in humans (70%) but may exhibit low bioavailability in pigs (23%). Similarly, the tetracycline group presents a low absorption fraction in pigs. Macrolides also reveal low bioavailability in humans (25–55%).

The metabolites are predominantly excreted as desmethyl-, acetyl-, carboxyl-, sulfoxide, and glucuronide conjugates. It should be highlighted that enrofloxacin is metabolised (20–50%) in the form of ciprofloxacin. Noteworthy to remark is the active metabolite 14-(R)-hydroxy-clarithromycin formed during clarithromycin biotransformation (Table 3).

## 3. Discussion

For the first time, a preliminary qualitative screening was performed in the Portuguese mainland to identify antibiotics in surface-ground water. The Polar Organic Chemical Integrative Sampler (POCIS) passive sampler and Ultra-performance Liquid Chromatography/Quadrupolo: Time-of-Flight Mass Spectrometry (UHPLC-QqTOF-MS) were used to monitor and analyse, respectively. This methodology is crucial for a preliminary selection of polar organic chemicals of concern which undergoes a diffuse emission into surface water due to continuous release into the environment [57,58,59]. As highlighted by Křesinová et al. [60], the main feature of this monitoring tool is the capacity to follow trace levels of organic contaminants in water bodies, demonstrating adequacy for assessing and managing the groundwater pollution. Several variables need to be considered in passive sampling: biofouling of surface, deployment strategy, and analyte(s) physicochemical properties. In our study, to obtain a view of environmental pressures, the sampling stations were in regions with urban/agricultural/rural impact for 30 days. This deployment time is considered ideal for the adsorption of the highest number of analytes [61]. 

This survey showed the occurrence of antibiotics in surface- groundwater samples in the Portuguese basin sampling sites, confirming the emission of these pharmaceuticals in the environment. Furthermore, antiviral drugs and enzyme inhibitors (beta-lactamase inhibitors and cilastatin) were also exhibited.

The relatively higher frequency detection of antibiotics downstream of the effluent discharge compared with detection in upstream samples obtained in rural locations was demonstrated and could be attributed to a low efficiency in the urban wastewater treatment plants or due to agricultural pressure.

The higher frequency of detection for most substances was observed in the Ave river and Ria Formosa, confirming that several effluents impact these water bodies from urban wastewater treatment plants and livestock production. 

Pharmacokinetic characteristics may represent key features in understanding antibiotics occurrence [62]. Most antibiotics are not completely metabolised in humans and animals; thus, a high percentage of the active substance (40–90%) is excreted in urine/faeces in the unchanged form. These molecules are discharged into water and soil through wastewater, animal manure, and sewage sludge, frequently used as fertilisers to agricultural lands. Also, it is expected that the hospital effluent will contribute partly to the pharmaceutical load in the wastewater treatment plant influence [63]. Afterwards, they may be transferred to groundwater by leaching [64]. 

Globally in the analysed samples, the most frequently detected antibiotics were fluoroquinolones (ciprofloxacin/enrofloxacin) and macrolides (clarithromycin/erythromycin) as tetracycline and sulfamethoxazole. 

In the present study, antibiotic metabolites were not detected in the water bodies. It may be related to their predominant excretion in conjugated form, rivalling or exceeding the parent compounds. These conjugates are presumed to be relatively hydrophilic, well excreted, and discharged into wastewater. Active and inactive metabolites within the wastewater treatment process may drawback [65] via deconjugation into the active substance by microbial enzymes (e.g., glucuronidases, sulfatases). 

Enrofloxacin was explicitly developed as veterinary medicine and introduced at the beginning of the 1980s. Thus, its detection suggests pollution sources from livestock farming, which explains why it was found in regions where animal production has prevailed. Ciprofloxacin is the primary degradation product of enrofloxacin. About 13–60% of enrofloxacin is metabolised into ciprofloxacin. Since ciprofloxacin is used in human medicine, its detection in the surface water is also due to wastewater discharge pollution. The fate of these fluoroquinolones in aquatic water bodies is dominated by adsorption (log Koc ≥ 4.2—strong sorption) and photodegradation reactions; they rapidly move from water to soil/sediments and onto organic particles solution, hence the occurrence in surface water. It has been reported that adsorption is more critical than photodegradation, which may be prominent when organic particles levels are low [31,66,67].

Nonetheless, besides the sorption capacity, these antibiotics have high solubility in water. Crucial routes for these substances into the environment are manure from animal production and sewage sludge from wastewater treatment plant (WWTP) used as fertilisers. Therefore, these substances have been evidenced in topsoil samples [68]. These quinolones and other antibiotics, for instance, norfloxacin and tetracycline, have been identified in groundwater samples despite being influenced by sorption processes. They were not readily degraded; instead, the input into groundwater could be due to livestock farming pressure, namely by spreading manure in the soil or the possible sewage sludge application in the area. High clay and low sand content in soils can decrease the mobility of pharmaceuticals, which is attributed to clay intense exchange capacity. Thus, soil properties (e.g. particle composition) are a significant, influential factor, especially relevant to groundwater, namely the input of pollutants [69]. Contrariwise, in our study, the wells were in podzolic soils/alluvial plain characterised by a sandy silt loam soil, favouring the antibiotics leaching to groundwater. In Portugal, in 2017, consumption of tetracyclines was six times higher than enrofloxacin plus ciprofloxacin. However, its frequency of detection in the surface water was similar, indicating differences in physicochemical behaviour, mainly the mobility feature in the environment (log Koc 4.9); studies demonstrated its high sorption capacity to clay materials, soil, and sediments [70]. As discussed above, for the fluoroquinolones (surface-groundwater), in a general sense, tetracycline seems to undertake a similar fate in the environment.

Nevertheless, due to stronger adsorption, with higher emergence in sediment, its occurrence in the surface water is lower [71]. The use of tetracyclines, mainly as medicated premix and oral solution for food-producing animals [72], and the very low bioavailability (e.g. in pig feed) [43] contribute to increasing its release into the environment. Regarding macrolides, erythromycin and clarithromycin exhibit a remarkable frequency of detection in surface water samples. The most frequently detected substance was clarithromycin (46%). Despite our small sampling number, the results follow the trend (58.8%) reported by Loos et al. [11]. In this EU report, azithromycin occurred at a frequency of 17.1% and erythromycin at 8.4%. In our study, the lowest detection frequency was found for azithromycin (8%), but erythromycin indicated higher values (38%). Although clarithromycin and azithromycin are exclusively used in human medicine, erythromycin is parenterally administered in farm animals. Considering the low human consumption, it is thus reasonable to assume that a considerable proportion of erythromycin in surface water may result from veterinary use. This substance has been detected in the Caia region, an area with significant agricultural pressure. 

The macrolide antibiotics (azithromycin and clarithromycin) demonstrated a potential tendency to bioaccumulate (log Kow values ≥ 3.0). Research on the determination of macrolide antibiotics in wastewater effluents concluded that they are not eliminated in WWTPs reaching the residual amounts of the surface water [73]. Thus, the removal of macrolides may result from a sorption process on activated sludge flocs and not directly from their biodegradation. However, due to their continuous consumption, it is frequently detected in surface water. Occurrence studies demonstrated that clarithromycin is one of the most frequently pharmaceutical in representative rivers [74,75]. The three macrolides identified in our detection survey are included since 2018 in the first “watch list” [76]. 

Another group of antimicrobial drugs identified in our survey were sulfamethoxazole/trimethoprim and sulfamethazine. Sulfamethoxazole/trimethoprim are often used combined since the effectiveness of sulfonamides is enhanced. In the present study, the detection of both substances was comparable; however, trimethoprim was detected in groundwater. They are moderately mobile and hydrophilic enough to be transferred into the aquatic environment (log Kow values ≤ 0.91). Different dissipation patterns in water and water:sediment systems were observed. Abiotic degradation and biotransformation were the main elimination pathway for sulfamethoxazole, being the degradation slightly increased in the presence of light, oxygen, and microbes.

Additionally, excreted sulphamethoxazole may contain approximately 30% to 70% acetylated conjugates [77]. The presence of acetylated metabolites such as N4-acetylsulfamethoxazole or N4-acetylsulfamethazine has been identified in WWTP effluents. Some of these metabolites can be converted back to the parent form [78,79]. In contrast to sulfamethoxazole, trimethoprim is not susceptible to photodegradation. The degradation rate was relatively slow, with half-lives around 30 days, explaining its occurrence in groundwater [20,80]. The possible pollution sources are from human and veterinary use. However, the environmental impact may be more critical due to active substances in drinking water or premix medicated feeds in veterinary medicine.

Sulfamethazine and lincomycin used only as veterinary medicines (cattle and swine production) were detected in a limited number of samples. Sulfamethazine was found once in a reservoir body, which presumably receives drain water from animal production. The relatively low sorption coefficients indicate that it is likely to be mobile in the aqueous runoff component following soil application [81]. Lincomycin in surface water was detected only in Algarve downstream from WWTP and was present in two wells. Due to moderate mobility [81], it could be mobilised from animal manure or animal production [82]. Lincomycin was found to leach to groundwater under environmental conditions encountered on the Canadian prairies, indicating that livestock manure management practices may result in antimicrobial surface and groundwater contamination [83]. 

Three factors may increase its occurrence in the environment: drinking water and premix for medicated feed and the poor oral absorption, and high excretion (79–86%) of the whole molecule through the faeces. Although the original goal was to detect antibiotics, it was observed that abacavir was the most frequently identified substance in our survey; for that reason and the recent academic interest in antiviral drugs, it was included in our study. Abacavir is used exclusively in human medicine in human immunodeficiency virus (HIV) infection treatment. Abacavir was detected in 11 of 13 surface sampling stations and one groundwater sampling station. Some studies demonstrated that abacavir is practically (>80%) removed from wastewater [84]. It undergoes phototransformation and biotransformation through oxidation of the terminal hydroxyl group to carboxy transformation products. Thus, abacavir was detected in influents while its carboxy-transformation products were the major components in effluents after transformation [84,85,86,87]. Studies concerning antiviral substances in the water bodies did not report the presence of abacavir in surface water and groundwater [88,89]. In our study, the detection of abacavir in surface-groundwater could be related to the weak efficiency of urban wastewater treatment plants. For the first time, as far as we know, clavulanic acid, cilastatin and tazobactam were detected in water bodies. Cilastatin inhibits dehydropeptidase, an enzyme found in the kidneys responsible for degrading antibiotics. Clavulanic acid and tazobactam are β-lactamase inhibitors both used in combination with antibiotics. Clavulanic acid is used in combination with amoxicillin, tazobactam with piperacillin/ceftolozane and cilastatin with the antibiotic imipenem. Piperacillin, ceftolozane and imipenem are antibiotics of exclusive hospital use [90]. Little is known about the behaviour of clavulanic acid and cilastatin in the environment. Tazobactam is a very high mobile hydrophilic substance; therefore, it could move quickly towards surface water. The most likely pollution source of these enzyme inhibitors is hospital wastewater. Cilastatin was recovered in groundwater from a well where most of the antibiotics were also found. This well was in a river basin in which a sandy silt loam soil predominates. 

All β-lactam antibiotics are rapidly degraded by biotic and abiotic processes [90]. However, in the present study, amoxicillin was detected in a sampling station, probably due to its high consumption (44 tonnes in human medicines). Besides its use in human medicine, amoxicillin is also widely used for treating and preventing animal diseases in many domestic and livestock animals. Several formulations of powder for administration in drinking water and medicated premix are available for poultry and pigs. The excretion of amoxicillin is predominantly renal; more than 80% of the parent drug is recovered unchanged in the urine. While bioavailability of 75 to 80% is reported in humans, a low value (~30%) was observed in pigs, calves, foals, and pigeons [26,52]. Maybe this last group of animals contribute more sharply to the release of this substance into the environment. In water, amoxicillin has a hydrolysis half-life of approximately 20 days (pH 7) [11,91]. Intermediate products generated from amoxicillin degradation are suspected to be more resistant to degradation and potentially more toxic than the parent compound [90,91]. The main problem related to the presence of this antimicrobial substance is the risk of inducing antimicrobial resistance genes [11,90]. Amoxicillin-S-oxide metabolite was reported as an oxidised degradation product derived by the indirect photolysis process. This β-lactam ring compound in aquatic environments may be of concern because it is still active [92]. Recently amoxicillin was included in the ’watch list’ in the Commission Implementing Decision (EU) 2018/840 [93].

A linkage between the detection frequency of antibiotics and their consumption was not established. However, the consumption amount is an essential complement for predicting a possible occurrence in the environment. It is reasonable to assume, from the obtained results, that various factors such as sorption and wastewater treatment influence the ratio occurrence/consumption [94]. Detailed information is missing on consumption data for veterinary medicinal products. However, considering the spot treatment approach, a trend between detection/consumption could be accepted locally.

As a concluding remark, we should emphasize that these substances have played a significant role in increasing life expectancy and the development of medical sciences. However, their prolonged use in agriculture and livestock industries, as a growth promoter, for treating animal infections, prophylaxis, and meta-prophylaxis has led to relevant antibiotic consumption worldwide. The use and misuse of antibiotics has stimulated the faster emergence of antibiotic-resistant bacteria and resistance genes, reducing their therapeutic potential against human and animal pathogens [94]. It is now clear that the environment is a vast reservoir of resistant organisms and their associated genes; thus, it is essential to understand environmental resistome and how its mobilisation into pathogenic bacteria may occur [94]. Several questions remain, but there is no doubt that exposure to antibiotics via agricultural practice and wastewater treatments are relevant contributing factors. In addition, microorganisms exposed to antibiotics at low, sub-lethal or sub-inhibitory exposure concentrations can develop, or acquire, antimicrobial resistance, which is now recognised as a significant threat to public health [95,96,97,98,99,100]. Contamination of surface water and groundwater by veterinary antimicrobials due to manure application is a cause of concern [101] regarding the emergence of antimicrobial-resistant bacteria in the water [83]. Additionally, the impacts of antibiotics on microbial communities and the abundance of resistance genes have also been described [102]. 

### The Need for the Ecopharmacovigilance

Research remains to be completed, focusing on either fate and ecotoxicological effects of these substances and the eco-resistance issue. In this regard, the occurrence of antibiotics/antivirals in water bodies raises concerns regarding the potential risk for all three components of the one health triad. As discussed before, the detected substances in water bodies may be related to their continuous release into the environment. These molecules belong to authorised medicinal products that have been consumed for several years. Their environmental risk assessment data is rudimentary or even missing.

The obtained results in this monitoring survey highlighted the imperative need to implement an ecopharmacovigilance framework. Holm et al. [103] reviewed, and similar to the pharmacovigilance system already implemented, eco-pharmacovigilance most significant challenge is the signal detection in the environment and the identification of cause-effect. Contrasting other pharmacotherapeutic groups, despite the complexity of detecting the cause-effect, the signal of bacterial resistance could be used to help manage this problem. 

In short, the understanding of resistance should be incorporated in a comprehensive Environmental Risk Assessment (ERA) and risk characterisation based not only on ecotoxicological endpoints but also on minimum selective concentrations (MSCs) endpoints. 

The current survey study is a powerful tool in the ecopharmacovigilance context. The results should be communicated, for instance, to healthcare professionals reinforcing the prudent use of these molecules. Moreover, considering the national specificity of such outcomes, recommendations should be included in antibiotic medicines regulation technical documents such as Summary Product Characteristics.

## 4. Materials and Methods

### 4.1. Study Area/Sampling Stations and Characterisation 

The study area (Portuguese mainland) comprised all river basin districts. Sampling campaigns were performed during 2017, 2018 and 2019 in four periods of 30 days each: March–April, April–May, May–June and Set-Out. 

**Figure 4:**
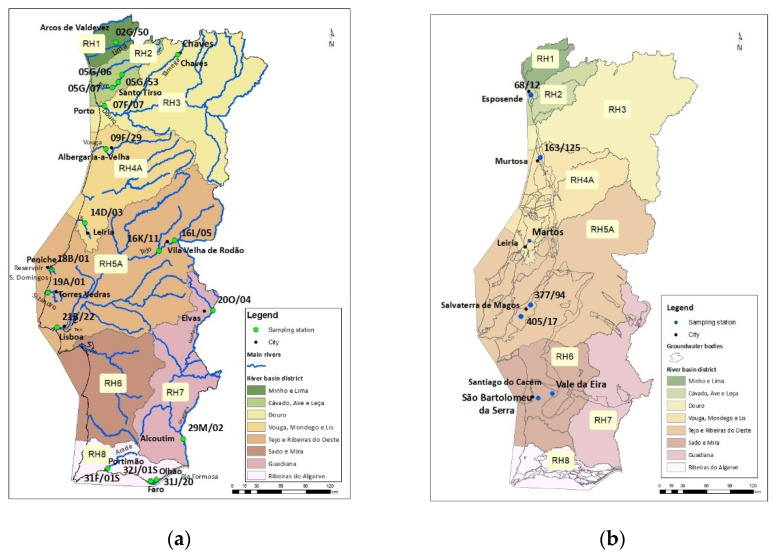
(**a**) Surface-water sampling sites; (**b**) groundwater sampling sites. Maps provided by the Portuguese Environmental Agency.

Eighteen sampling stations were in the main river basins and their tributaries, reservoirs, transitional waters, and coastal waters for surface-water monitoring. In each of them, a passive sampling system was placed 50 cm deep into the water column during the periods of reduced river flow rate and hold in place for approximately 30 days. However, it was only possible to collect samples from 13 stations due to the damage of the passive sampler in the remaining ones. Groundwater samples were collected in wells from seven sampling stations. The placement of passive samplers in groundwater considered the well technical features; the depth and groundwater level were previously determined since they should be detected at the superficial levels. The passive sampler was placed using a water level meter, 2 m below the groundwater level. The sampler always remained immersed in water, avoiding extractions and the regional lowering of the water table [104]. For the sampling stations, sites of different environmental pressures were considered, specifically urban, agricultural area/animal production, and aquaculture. The information regarding the potential risks of each source was obtained on the River Basin Management Plans and the CORINE Land Cover 2018 [105]. The sampling sites are shown in Figure 4a,b.

### 4.2. Materials and Chemicals

#### 4.2.1. Passive Sampler Field Deployment

The passive sampler Polar Organic Chemical Integrative Sampler (POCIS) used in this study is a hydrophilic-lipophilic balance (HLB) polymer of divinylbenzene vinylpyrrolidone enclosed between two polyether sulphone (PES) membranes. This PES has the advantage of little biofouling, allows the qualitative detection of many organic compounds in the dissolved phase, including antibiotics. The field deployment uses, for surface waters, a canister, and inside two individual POCIS units were performed to protect each POCIS. In rivers, the column of water deployment was done with medium or low flows; otherwise, POCIS might be damage. In ground-waters was used one POCIS unit/well. Due to the high sorption capacity, POCIS was deployed approximately for 30 days, allowing the polar organic compounds adsorbed to be in the equilibrium stage with the active substances in an aqueous medium. In the laboratory, POCIS disks were frozen until extraction.

#### 4.2.2. Qualitative Analysis Method Used for the Characterisation of Antibiotics in Surface-Groundwater

This methodology involved two steps corresponding to the extraction of the adsorbent of POCIS extraction disks and subsequent qualitative determination by Ultra Performance Liquid Chromatography-High Resolution Mass Spectrometry (UHPLC-QqTOF-MS).

##### Sample Extraction 

After drying the POCIS disks, the membrane on one side of the support was cut. The adsorbent was transferred to an empty cartridge, extracted five times with 2 mL of methanol, making a total volume of 10 mL collected in a glass vial. This extract was concentrated under nitrogen current to a final volume of about 1 ml. To the extract was added the ^13^C_3_ internal caffeine standard. The extract in methanol is taken to dryness in rotavapor under vacuum at 35 °C and reconstituted in a mixture of water/methanol (v:v 4:1) for subsequent injection into the UHPLC-QqTOF-MS system. For surface water samples, two discs for sampling collection were analysed. The adsorbents of the duplicated discs were extracted simultaneously. In the case of groundwater samples, only one POCIS sampler was used per collection point.

##### Ultra-Performance Liquid Chromatography-High Resolution Mass Spectrometry (UHPLC-QqTOF-MS) Analysis

Extracts were analysed on a UHPLC-QqTOF-MS system composed of an UltiMate^®^ 3000 RSLCnano system (Thermo Scientific Inc., Waltham, MA, USA), interfaced with a QqTOF Impact II mass spectrometer with an ESI source (Bruker Daltonics, Bremen, Germany). Three aliquots were analysed in both ESI positive and negative mode for each water extract, and as a control, a “blank” sample of the mixture water/methanol (4:1).

Chromatographic separation was achieved with a Kinetex C18 column (150 × 2.1 mm; 2.6 μm particle size; Phenomenex, Torrance, CA, USA), using an elution gradient of 0.1% *v*/*v* formic acid in water (mobile phase A) and 0.1% *v*/*v* formic acid in acetonitrile (mobile phase B), at a flow rate of 200 μL/min. The elution conditions were as follows: 0–1.4 min, isocratic 7% B; 1.4–10.0 min, linear gradient to 50% B; 10–15 min, linear gradient to 100% B; 15–18 min isocratic 100% B; 18–19 min, linear gradient to 7% B; 19–25 min, isocratic to 7% B. The injection volume was 20 µL. The column and the autosampler were maintained at 35 °C and 8 °C, respectively. The mass spectrometer parameters were set as follows: endplate offset: 500 V; capillary voltage: 4.5 and 2.5 kV (positive and negative mode, respectively); nebuliser: 2.8 bars; dry gas: 8 L/min; dry temperature: 200 °C. Internal calibration was performed for sodium formate clusters, with a sodium formate solution 10 mM introduced to the ion source via a 20 µL loop, at the beginning of each analysis using a six-port valve. Calibration was then performed using high-precision calibration mode (HPC). The mass spectra were acquired in the broadband collision-induced dissociation (bbCID) mode in a range between 50–1000 *m/z*, with a scan speed of 1 Hz. MS/MS experiments were performed in a data-dependent-acquisition (DDA) mode with an acquisition rate of 3 Hz using a dynamic method with a fixed cycle time of 3 s. Dynamic exclusion duration was 0.4 min. The 49 standards were analysed in the MRM acquisition mode, with collision energy defined according to the mass, load of each ion and an isolation window of 5 Da. Under the instrumental conditions used, the TOF (FWHM) resolution power is between 36,000–40,000 to *m/z* 226.1593, 430.9137 and 702.8636. 

#### 4.2.3. Data Analysis and Validation

Mass spectrometry data were processed using the Data Analysis 4.4 and Target Analysis 1.3 software (Bruker Daltonics). An in-house mass library was built for 49 standards plus 620 suspected substances (insecticides, fungicides, herbicides, drugs, metabolites). For the 49 standards previously analysed by MRM, the database includes the expected retention time, accurately measured mass, molecular formula, and qualifier ions for each standard compound. The (possible) elemental composition of peaks of interest was calculated using the algorithm Smart Formula 3D within Bruker software for the remaining compounds. This library was used for accurate mass screening for (non)target compounds and unknowns using the Find-Compounds-Chromatogram via SigmaFiTTM tools of the Target Analysis software, which creates a “peak chromatogram base” for *m/z* values above a threshold intensity value. Values for retention time deviation lower than 0.3 min; mass deviation less than 5 ppm and mSigma less than 100 were considered acceptable for positive confirmation (mSigma < 100, acceptable, <50 good, and <25 excellent). After screening in Target Analysis, the Data Analysis potentialities were then used to check the data manually. Analysing the samples with a QqTOF mass spectrometer, the pharmaceuticals in complex matrices were detected at concentrations as low as 50 ng/L.

#### 4.2.4. Chemicals and Reagents

Methanol HPLC Ultragradient Gold, formic acid 99% and ammonia solution 25% were purchased from Carlo Erba Reagents, SAS (Marseille, France). H_2_O - Pierce™. Water, acetonitrile and formic acid LC-MS grade Optima^®^ were from Fisher Chemical (Hampton, NH, USA), state abbrev if USA, country). Caffeine-^13^C_3_ (99%, 1 mg/mL in methanol) was obtained from Sigma-Aldrich (Schnelldorf, Germany). Standards (purity ≥97%) of all the chemicals listed (*tert*-butylazine; desethyl-*tert*-butylazine; dimethoate; atrazine; desethylatrazine; tebuconazole; alachlor; omethoate; bentazone; diuron; linuron; chlorotoluron; cymoxanil; thiamethoxam; clothianidin; imidacloprid; acetamiprid; thiacloprid; simazine; isoproturon; cybutryne; methiocarb; quinoxyfen; chlorpyrifos; 2,4D; MCPP; aclonifen; bifenox; MCPA; dichlorvos; bezafibrate; ibuprofen; amoxicillin·3H_2_O; azithromycin; diclofenac; α-ethinylestradiol; β-estradiol; erythromycin; clarithromycin; trimethoprim; ciprofloxacin; enrofloxacin; sulfamethoxazole; bisoprolol; carbamazepine) were purchased from Sigma-Aldrich.

### 4.3. Consumption of Detected Antibiotics

Information on pharmaceuticals sales data (corresponding to 2017) by the package, pharmaceutical form, and quantitative composition relative to the detected antibiotics, were obtained from the Department for the Medicine’s Economic Assessment of the INFARMED, thus allowing estimation of the amount of active substance for each compound [106]. These substances comprise the exclusive hospital use medicines and human primary health care sector. The data of antibiotics explicitly used in farm animals were gathered on the ESVAC report [107]. 

### 4.4. Physicochemical Properties and Key Pharmacokinetic Features of Detected Antibiotics 

A review focused on the primary physicochemical and pharmacological characteristics was performed to interpret the possible correlations between the detected and consumed antibiotics. Pharmacokinetic features of antibiotics, particularly the proportion of excretion as the parent compound and bioavailability, may contribute to a major or lesser environmental impact related to the corresponding reported occurrence in different environmental compartments [62]. Thus, besides degradation products found in the environment, knowledge of metabolic reactions in the human body and the treated animal was considered. Metabolites will be herein referred to as the molecules resulting from biotransformation of the chemical structure within the body of humans and treated animals. The main pharmacokinetic features were summed up in Table 3. The physicochemical properties were gathered up in Table 2.

## 5. Conclusions

The passive sampler (POCIS) allows an increase of sensitivity not achievable with grab sampling monitoring in surface water and groundwater. The detection and the unequivocal identification of antibiotics/enzyme inhibitors and antiviral substances were performed with liquid chromatography-high resolution mass spectrometry.

The most frequently detected active substances in surface water were as follows: enrofloxacin/ciprofloxacin, clarithromycin/erythromycin, tetracycline, sulfamethoxazole, and abacavir. In groundwater, enrofloxacin/ciprofloxacin, norfloxacin, trimethoprim, lincomycin, abacavir and tetracycline were recovered. Metabolites were not detected in water bodies. Noticeable was the detection of enzyme inhibitors, tazobactam and cilastatin, which are both for exclusive hospital use. The North region and Algarve (South) were the areas with the most significant frequency of substances in surface water. The relatively higher detection of substances downstream of the effluent discharge points compared with a low detection in upstream samples could be attributed to the low efficiency in urban wastewater treatment plants or agricultural pressure. The environmental impact is more critical due to active substances in drinking water or premix medicated feeds in the veterinary site.

Furthermore, the detection of substances of exclusive human use (abacavir, tazobactam and cilastatin) prove the weak efficiency of urban wastewater treatment plants. Groundwater contamination was demonstrated upon the presence of substances in wells. A linkage between the detection frequency of detected substances and their consumption was not established. 

This screening approach is essential not only to identify substances in order to perform further quantitative environmental risk assessment but also to establish possible water quality standards and confirm the classification of these molecules as possible priority or priority hazardous substances. 

The greatest challenge of this survey data will be to promote an ecopharmacovigilance framework, implementing measures to avoid misuse/overuse of antibiotics and slow down emission and antibiotic resistance. The information and the analysis provided in this work has highlighted the imperative need to implement such an ecopharmacovigilance framework.

## Figures and Tables

**Table 1:** Consumption (Kg) of the detected pharmaceuticals in Portugal (2017).

Pharmaceuticals	Regional Amount (Kg)					Σ
	North	Centre	West/Tejo	Alentejo	Algarve	
Antibiotics						
Amoxicillin	15,494	9664	15,669	1715	1866	44,408
Enrofloxacin	NA	NA	NA	NA	NA	3600 ^1^
Ciprofloxacin	1075	744	1370	140	154	3483
Norfloxacin	77	69	155	22	17	340
Sulfamethoxazole	796	589	1002	95	47	2529
Sulfamethazine	NA	NA	NA	NA	NA	5800 ^1,2^
Erythromycin	32	27	59	5	7	130
Azithromycin	536	393	659	64	77	1729
Clarithromycin	630	620	800	140	40	2230
Trimethoprim	159	118	200	19	41	537
Tetracycline	NA	NA	NA	NA	NA	45,000 ^1^
Lincomycin	NA	NA	NA	NA	NA	3000 ^1^
Inhibitors						
Clavulanic acid	1535	989	1587	175	188	4474
Tazobactam	228	159	279	29	22	717
Cilastatin	57	13	5	0.6	0.3	76
Antiviral						
Abacavir	430	116	848	17	47	1458

^1^ Amount from ESVAC Report-2017; ^2^ Match the sulfonamides amount; NA—not available.

**Table 2:** Physicochemical properties of detected pharmaceuticals.

Active Substances	CAS-N° ^1^	MW ^1^ g/mol	Water Solubility ^1^	pKa Acid and Basic ^1^	log Kow ^1^	log Koc L/kg
Amoxicillin	26787-78-0	365.4	high	7.4	0.87	3.3 ^2^
Enrofloxacin	93106-60-6	359.4	high	6.7	0.58	4.22–5.89 ^3^
Ciprofloxacin	85721-33-1	331.3	high	6.1	0.28	4.78 ^3^
Norfloxacin	70458-96-7	319.3	high	5.8	−1.03	4.4 (Kd) ^4^
				8.7		
Sulfamethoxazole	723-46-6	253.3	high	6.2	0.89	2.3 ^5^
				2		
Sulfamethazine	57-68-1	278.3	high	7.6	0.89	1.78–2.32 ^3^
Erythromycin	114-07-8	733.9	low	8.9	2.6	2.3 ^6^
Azithromycin	83905-01-5	749	high	8.5	3	3.5 ^7^
Clarithromycin	81103-11-9	748	high	9	3.2	2.2 ^8^
Trimethoprim	738-70-5	290.3	moderate	7.1	0.91	2.5 ^5^
Tetracycline	60-54-8	444.4	low	3.3	−1.3	4.9 ^9^
Lincomycin	859-18-7	406.5	high	7.6	0.56	NA
Tazobactam	89786-04-9	300.3	high	2.1	−1.8	0.87 ^10^
Clavulanic acid	58001-44-8	199.2	high	2.7	−2.3	NA
Cilastatin	82009-34-5	358.5	moderate	9.5	0.29	NA
				2.5		
Abacavir	136470-78-5	286.3	high	15.4	1.2	3.0 ^6^
				5.8		

^1^ Data retrieved from [16]; ^2^ Data retrieved from [17]; ^3^ Data retrieved from [18]; ^4^ Data retrieved from [19]; ^5^ Data retrieved from [20]; ^6^ Data retrieved from [21]; ^7^ Data retrieved from [22]; ^8^ Data retrieved from [23]; ^9^ Data retrieved from [24]; ^10^ Data retrieved from [25]; NA—not available.

**Table 3:** Main pharmacokinetic properties of detected pharmaceuticals.

Active Substance	Therapeutical Use Dosage Forms ^1,2^—Target Species	Bioavailability ^1,2^	Excretion		References
			Unchanged Form	Metabolites (%)	
Amoxicillin	Human use Capsule; tablets; Powder for oral suspension; Powder for solution for injection or infusion. Veterinary use Premix for medicated feed—pigs; Powder in drinking water—poultry, pigs; Injectable suspension—cattle, sheep, pigs; Palatable tablets—dogs and cats.	70%; 23% ^3^	Renal: 80–90% Faecal: 5–10%	Amoxicilloic acid; Piperazine-2,5-dione (diketopiperazine). (Both 10–20%)	[26,27,28,29]
Enrofloxacin	Veterinary use Oral solution—cats, pigs, broilers, rabbits Solution for drinking water—chickens, turkeys, rabbits; Solution for injection—dogs, cats, cattle, pigs; Tablets—dogs and cats.	73–101% ^3^	Renal: 21% ^3^ Faecal: 19% ^3^	Ciprofloxacin (20–50%); Active dealkylated and hydroxylated enrofloxacin (<10%).	[30,31]
Ciprofloxacin	Human use Tablets.	64–85%	Renal: 30–50% Faecal: 15–62%	Oxociprofloxacin; Desethylene ciprofloxacin; Formylciprofloxacin.	[32,33]
Norfloxacin	Human use Tablets; Eye drops solution.	30–50%	Renal: 30–70% Faecal: 30%	3-oxo-1-piperazinyl metabolite (<20%).	[34]
Sulfamethoxazole	Human use ^5^ Tablets; Syrup; Solution for injection. Veterinary use ^4^ Solution for drinking water—pigs.	NA	Renal: 10–40%	N4-acetyl- sulfamethoxazole (30–70%); Sulfamethoxazole glucuronide.	[35]
Sulfamethazine (sulfadimidine)	NA	NA	Renal: <12%	N4-acetyl-sulfamethazine (61–81%); Conjugated hydroxylated Metabolites (10–20%).	[36]
Erythromycin ^5^	Human use Tablets; Granules for oral suspension; Powder for solution for injection; Topical skin solutions. Veterinary use Injectable solution—cattle, pigs and sheep.	25%	Renal: 2–5%	N-desmethyl-erythromycin; Anhydroerythromycin.	[37,38]
Azithromycin	Human use Powder for oral suspension; Powder for solution for infusion; Tablets; Eye drops solution.	37%	Renal: 20%	N-desmethyl derivatives.	[37,38]
Clarithromycin	Human use Tablets; Granules for oral suspension; Powder for solution for injection.	55%	Renal: 30–40%	14-(R)-hydroxy-clarithromycin (active metabolite); N-demethyl-clarithromycin.	[37,38,39]
Trimethoprim	Human use ^6^ Tablets; Syrup; Solution for injection; Veterinary use ^6^ Premix for medicated feed—pigs and sheep; Solution for drinking water (pigs, broilers, calves, lambs, rabbits); Solution for injection—cattle, horses, sheep; Oral paste and oral powder—horses.	90% ^3^	Renal: ±80%	3-Desmethyl-trimethoprim (65%); 4-Desmethyl-trimethoprim (25%); N-oxides (≤5%).	[40,41]
Tetracycline	Human use Capsule; Ophthalmic ointment; Veterinary use Premix for medicated feed—fish farm; pigs. Powder for oral solution—calves, lambs, pigs, rabbits and poultry; Solution for injection—cattle, pigs, horses, sheep, dogs and cats.	5% ^3^	Renal: 30% Faecal: 20–60%	D-epitetracycline (5%).	[42,43]
Lincomycin	Veterinary use Premix for medicated feed—pigs; Powder for drinking water—pigs, chickens; Injectable solution—cattle, sheep, goats, swine, chickens, turkeys, cats; Intramammary solution for lactating cows.	20–50% ^3^	Renal: 14–21% ^3^ Faecal: 79–86% ^3^	Lincomycin sulphoxide.	[44,45]
Tazobactam	Human use ^7^ Powder for solution for injection or infusion.	NA	Renal: 60–80%	M1-hydrolyzed metabolite (20–26%).	[46,47,48]
Clavulanic Acid ^4^	Human use ^8^ Tablets; Powder for oral suspension; Powder for solution for injection or infusion. Veterinary use Powder for drinking water—pigs; Powder for oral suspension—dogs and cats; Injectable—Cattle, Canines, Felines, Pigs; Palatable tablets—dogs and cats; Intramammary suspension for lactating cattle.	45% ^3^	Renal: 40–73%	2,5-dihydro-4-(2- hydroxyethyl)-5-oxo-1H-pyrrole-3-carboxylic acid (15.6%). 1-amino-4-hydroxy-butan-2-one (<10%).	[26,49,50,51,52]
Cilastatin	Human use ^9^ Powder for solution for infusion.	NA	Renal: 78%	N-acetyl metabolite (10%).	[53,54]
Abacavir ^5^	Human use Film-coated tablet ^10^; oral solution.	83%	Renal: 1% Feacal: 16%	5′-carboxylic-acid metabolite (30%); 5′-glucuronide metabolite (36%); Minor metabolites (15%).	-

^1^ Data retrieved from Infomed—National database for human use [55]; ^2^ Data retrieved from MedVet—Nacional database for veterinary use [56]; ^3^ Depicted data is from pigs; ^4^ Associated to trimethoprim; ^5^ Some data retrieved from Drugbank database [16]; ^6^ Associated to sulfamethoxazole and other sulonamides; ^7^ Associated to piperacillin/ceftolozane; ^8^ Associated to amoxicillin; ^9^ Associated to imipenem; ^10^ Associated to lamivudine; NA = Not available.

## Supplementary Materials

The following are available online at https://www.mdpi.com/article/10.3390/antibiotics10080888/s1, Table S1. Surface-water: characterisation of the sampling stations; Table S2. Groundwater: characterisation of the sampling stations; Table S3. Detection of frequency and geographic distribution of Pharmaceuticals in Surface Water (2017_2018_2019) March-April, April-May, May-June and Set-Out; Table S4. Detection of frequency and geographic distribution of Pharmaceuticals in groundwater (2017_2018_2019).
