# Supplementary material for: Identification of Antibiotics in Surface-Groundwater. A Tool towards the Ecopharmacovigilance Approach: A Portuguese Case-Study
Source: Antibiotics (Basel). 2021 Jul 21;10(8):888. doi: 10.3390/antibiotics10080888 (PMC8388677; doi:10.3390/antibiotics10080888)
Supplement: Supplementary file 1 [file antibiotics-10-00888-s001.zip › Table S1. Surface-water characterization of the sampling stations..pdf]

Table S1. Surface-water: characterization of the sampling stations.

Regions	River basin district (HR)	Water bodies	Sampling station	station code /geographic coordinates	Localisation	WWTP[1] equivalent inhabitants	Potential Pressures
North	HR2	Ave river	1	PT05G/06 Lat. (ºN): 41,49565 Long (ºW): -8,3212	Downstream of Prazins	-	Agriculture / Animal production
			2	PT05G/53 Lat. (ºN): 41,40951 Long (ºW): -8,38342	Downstream of UWWTP (Guimarães) Serzedelo I and II.	Serzedelo I:126.000 Serzedelo II: 270.822	Urban Agriculture / Animal production
	HR3	Tâmega (international river)	3	Chaves Lat. (ºN): 41,713199 Long (ºW): -7,507799	Downstream of UWWTP (Chaves). A tributary (sub-basin) of Douro river	Chaves: 57.748	Urban Agriculture / Animal production
		Tinto river	4	PT07F/05 Lat. (ºN): 41,1539428 Long (ºW): -8,5702716	Rio Tinto-Campanhã Downstream of UWWTP. A tributary (sub-basin) of Douro river	Freixo: 170.000	Urban
Center	HR4A	Vouga river	5	PT09F/29 Lat. (ºN): 40,672777 Long (ºW): -8,560407	Downstream of Angeja	-	Agriculture / Animal production
West and Tejo Region	HR5A	S. Domingos (west - (reservoir)	6	PT18B/01 Lat. (ºN): 39,33406 Long (ºW): -9,31713	S. Domingos reservoir	-	Agriculture / Animal production
		Tejo (international river)	7	PT16L/05 Lat. (ºN): 39,660322 Long (ºW): -7,569093	Perais, near the border Portugal /Spain	-	Rural
			8	PT16K/11 Lat. (ºN): 39,5474596 Long (ºW): -7,7934542	Fratel reservoir	-	Rural
			9	PT21B/22 Lat. (ºN): 38,69522 Long (ºW):	Marina Algés, transitional water,	Beirolas: 213.500 Chelas: 255.000	Urban, Agriculture

				-9,234269	downstream of<br>3 UWWTP:	Alcântara: 756.000	
Alentejo	HR6 /HR7	Caia river	10	PT20O/04<br>Lat. (ºN): 38,8831<br>Long (ºW): -7,0356	Herdade da Comenda, near the border Portugal /Spain	-	Agriculture / Animal production
Algarve	HR8	Ria Formosa (Coastal water)	11	PT31J/02<br>Lat. (ºN): 37,00194<br>Long (ºW): -7,92199	Ria Formosa –Faro, Downstream of UWWTP	– Faro / Northwest 44.530	Urban
			12	PT31J/20<br>Lat. (ºN): 37,01814<br>Long (ºW): -7,84396	Ria Formosa - Olhão Downstream of UWWTP	Faro / Olhão: 113.200	Urban<br>Fish stocking<br>Fishing activity
		Arade (Transitional water)	13	PT31F/01S<br>Lat. (ºN): 37,14425<br>Long (ºW): -8,51528	Portimão - UWWTP	Portimão: 140.000	Urban

[1]Waste Water Treatment Plants
