# Supplementary material for: Identification of Antibiotics in Surface-Groundwater. A Tool towards the Ecopharmacovigilance Approach: A Portuguese Case-Study
Source: Antibiotics (Basel). 2021 Jul 21;10(8):888. doi: 10.3390/antibiotics10080888 (PMC8388677; doi:10.3390/antibiotics10080888)
Supplement: Supplementary file 1 [file antibiotics-10-00888-s001.zip › Table S2. Groundwater characterization of the sampling stations.pdf]

Table S2. Groundwater: characterization of the sampling stations.

Regions	River basin district (RH)	Sampling station	station code /geographic coordinates	Localisation (well)	Potential Pressures
North	RH2	1	68/12<br>41.490;<br>-8.744	Esposende	Agriculture<br>Animal production
Center	RH4A	2	163/125<br>40.767;<br>-8.599	Murtosa	Urban<br>Agriculture<br>Animal production
		3	-<br>39.808;<br>-8.751	Martos	Agriculture<br>Animal production
West and Tejo Region	RH5A	4	405/17<br>38.939;<br>-8.863	Samora Correia	Agriculture<br>Animal production
		5	377/94<br>39.073;<br>-8.728	Salvaterra de Magos	Agriculture<br>Animal production
Alentejo	RH6/HR7	6	-<br>38.052;<br>-8.399	Monte da Horta /Vale da Eira	Agriculture<br>Animal production
		7	-<br>37.996;<br>-8.607	São Bartolomeu da Serra	Agriculture<br>Animal production
