# Supplementary material for: Identification of Antibiotics in Surface-Groundwater. A Tool towards the Ecopharmacovigilance Approach: A Portuguese Case-Study
Source: Antibiotics (Basel). 2021 Jul 21;10(8):888. doi: 10.3390/antibiotics10080888 (PMC8388677; doi:10.3390/antibiotics10080888)
Supplement: Supplementary file 1 [file antibiotics-10-00888-s001.zip › Table S3. Frequency detection and geographic distribution_Surface-water_updated.pdf]

Table S3. Frequency detection and geographic distribution of pharmaceuticals in surface water (2017_2018_2019).

		**Period**	2017					2018					2019		
		**Season**	March April			May June		April May		May June		Sep October	April May	July	
		**Water bodies**	Tejo	Tejo	Vouga	Caia	Ria Formosa	Ave	Ave	Arade	Ria Formosa	Tejo	Tâmega	Rio Tinto	S.Domingos	
		**Station Nr. (n=13)**	8	7	5	10	12	1	2	13	11	9	3	4	6	Frequency
**Pharmacoterapeutical Group**		**Active Substance**														(d/n)x100 %[1]
**Antibiotics**	Penicillins	Amoxicillin			X									X		15
	Quinolones	Enrofloxacin				X	X	X			X					31
		Ciprofloxacin			X	X	X	X		X	X					46
		Norfloxacin							X							8
	Sulphonamides	Sulfamethoxazole			X			X						X		23
		Sulfamethazine													X	8
	Macrolides	Erythromycin				X		X	X				X	X		38
		Azithromycin					X									8
		Clarithromycin					X	X	X		X		X	X		46
	Trimethoprim	Trimethoprim								X		X				15
	Tetracyclines	Tetracycline			X			X			X			X		31
	Lincosamides	Lincomycin								X	X					15
**Beta-lactamase inhibitors**		Clavulanic acid				X								X		15
		Tazobactam							X		X					15
**Antiviral**		Abacavir			X	X	X	X	X	X	X	X		X		69
		**Geographic distribution**	0	0	5	5	5	7	5	4	7	2	2	7	1	**∑ 50 detected ative substances**
		**Region**	West/Tejo	West/Tejo	Centre	Alentejo	Algarve	North	North	Algarve	Algarve	West/Tejo	Center	North	West/ Tejo	North:19 – 38%<br>Algarve:16 – 32%<br>Centre:7 – 14%<br>Alentejo:5 – 10%<br>West/Tejo:3 – 6%

[1] Frequency=(d/n)x100, where *d*=number of detections and *n*=number of stations.
