# Supplementary material for: Identification of Antibiotics in Surface-Groundwater. A Tool towards the Ecopharmacovigilance Approach: A Portuguese Case-Study
Source: Antibiotics (Basel). 2021 Jul 21;10(8):888. doi: 10.3390/antibiotics10080888 (PMC8388677; doi:10.3390/antibiotics10080888)
Supplement: Supplementary file 1 [file antibiotics-10-00888-s001.zip › Table S4. Frequency detection and geographic distribution_Groundwater_updated.pdf]

Table S4. Detection of frequency and geographic distribution of pharmaceuticals in groundwater (2017_2018_2019).

		**Period**	2017			2018		2019		
		**Season**	March April			May June		April May		
		**Station Nr.**	1	2	3	4	5	6	7	
**Pharmacoterapeutical Group**		**Active Substances**								Frequence (n=7) (d/n)x100 %[1]
**Antibiotics**	Quinolones	Ciprofloxacin	**X**	**X**			**X**			43
		Enrofloxacin		**X**			**X**			29
		Norfloxacin				**X**		**X**		29
	Trimethoprim	Trimethoprim		**X**			**X**			29
	Tetracyclines	Tetracycline					**X**			14
	Lincosamides	Lincomycin				**X**			**X**	29
**Beta-lactamase inhibitors**		Clavulanic acid		**X**						14
**Renal Dehydropeptidase-I inhibitor**		Cilastatin		**X**						14
**Antiviral**		Abacavir			**X**					14
		Geographic distribution	1	5	1	2	4	1	1	∑ 15 detected active substances
		Region	North	Centre	Centre	West /Tejo	West /Tejo	Alentejo	Alentejo	West/Tejo: 6 – 43%<br>Centre: 6 – 43%<br>Alentejo: 2 – 14%<br>North: 1 – 7%

[1]Frequency = (d/n)x100, where *d*=number of detections and *n*=number of stations.
